# Bickerstaff’s brainstem encephalitis, Miller Fisher syndrome and Guillain-Barré syndrome overlap in an asthma patient with negative anti-ganglioside antibodies

**DOI:** 10.1186/1756-0500-5-295

**Published:** 2012-06-14

**Authors:** Chongyu Han, Yuan Wang, Jianping Jia, Xunming Ji, Vance Fredrickson, Yuchuan Ding, Wei Sun, Jia Xu, Yong-Xin Sun

**Affiliations:** 1 Department of Neurology, You Anmen Hospital, 100069, Beijing, People's Republic of China; 2 Department of Neurology, Xuan Wu Hospital of Capital Medical University, 100053, Beijing, People's Republic of China; 3 Department of Neurological Surgery, Wayne State University School of Medicine, Detroit, MI, 48201, USA

**Keywords:** Asthma, Autoimmune, Bickerstaff’s brainstem encephalitis, Miller Fisher syndrome, Guillain-Barré syndrome

## Abstract

**Background:**

Bickerstaff’s brainstem encephalitis (BBE), together with Miller Fisher syndrome (MFS) and Guillain-Barré syndrome (GBS) were considered to form a continuous clinical spectrum. An anti-GQ1b antibody syndrome has been proposed to underlie the common pathophysiology for the three disorders; however, other studies have found a positive anti-GM1 instead of anti-GQ1b antibody.

**Case presentation:**

Here we report a 20-year-old male patient with overlapping BBE, MFS and GBS. The patient had a positive family history of bronchial asthma and had suffered from the condition for over 15 years. He developed BBE symptoms nine days after an asthma exacerbation. During the course of illness, he had significantly elevated IgE levels in both serum and cerebrospinal fluid. Serologic analysis of antibodies against ganglioside complexes (anti-GDIa, anti-GDIb, anti-GM1, anti-GM2, anti-GM3, anti-GQIb and anti-GTIb antibodies) showed negative results.

**Conclusions:**

Since asthma has recently been related to autoimmune disease, our case supports an autoimmune mechanism underlying the clinical spectrum composed of BBE, MFS and GBS. However, contrary to a proposed anti-GQ1b antibody syndrome, we would suggest that pathogenesis of this clinical spectrum is not limited to anti-ganglioside antibodies.

## Background

Patients with overlapping Bickerstaff’s brainstem encephalitis (BBE), Miller Fisher syndrome (MFS) and Guillain-Barré syndrome (GBS) were rarely reported outside of Japan. The three disorders have been considered part of a clinical spectrum, however, a common underlying pathophysiology is still being investigated [1]. An anti-GQ1b antibody syndrome has been proposed to associate BBE, MFS, GBS and other similar conditions [2]. Despite this proposed anti-GQ1b syndrome, a positive anti-GM1 antibody has also been demonstrated in an overlapping case of BBE, MFS and GBS rather than the expected anti-GQ1b [3]. Here, we report a case of overlapping BBE, MFS and GBS, in which all tested ganglion nucleoside antibodies were negative, serum IgE showed significant elevation, and a positive family history of bronchial asthma was present. Most recently, studies have suggested that asthma has an autoimmune pathogenesis similar to various autoimmune diseases [4]. The patient displayed BBE, MFS and GBS as a continuous clinical course related to an autoimmune response. Since various autoimmune mechanisms have been suggested for asthma [5] a clinical syndrome composed of BBE, MFS and GBS may have a broader immunologic basis rather than a single autoantibody-mediated response against a ganglioside complex.

## Case presentation

### Case report

**Figure 1:**
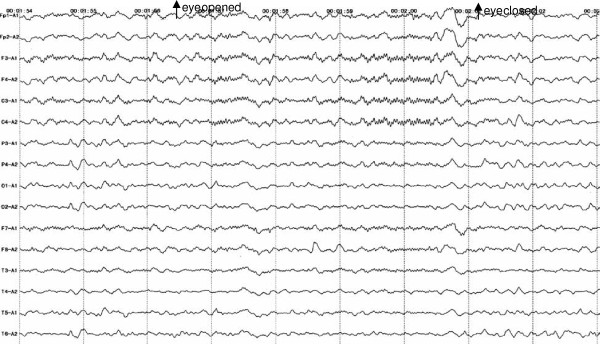
**Electroencephalography pattern from 20-year-old patient with overlapping Bickerstaff’s brainstem encephalitis, Miller Fisher syndrome, and Guillain-Barré syndrome.** A diffuse slow wave EEG pattern with 4-6 cycles per second is seen in all electrodes. Twenty-two to twenty-six cycles per second waveform predominates over the centroparietal area, bilaterally. The alpha rhythm over the occipital electrodes was absent. By the time of examination, the patient’s consciousness level demonstrated intermittent drowsiness, suggesting a diagnosis of encephalitis.

**Figure 2:**
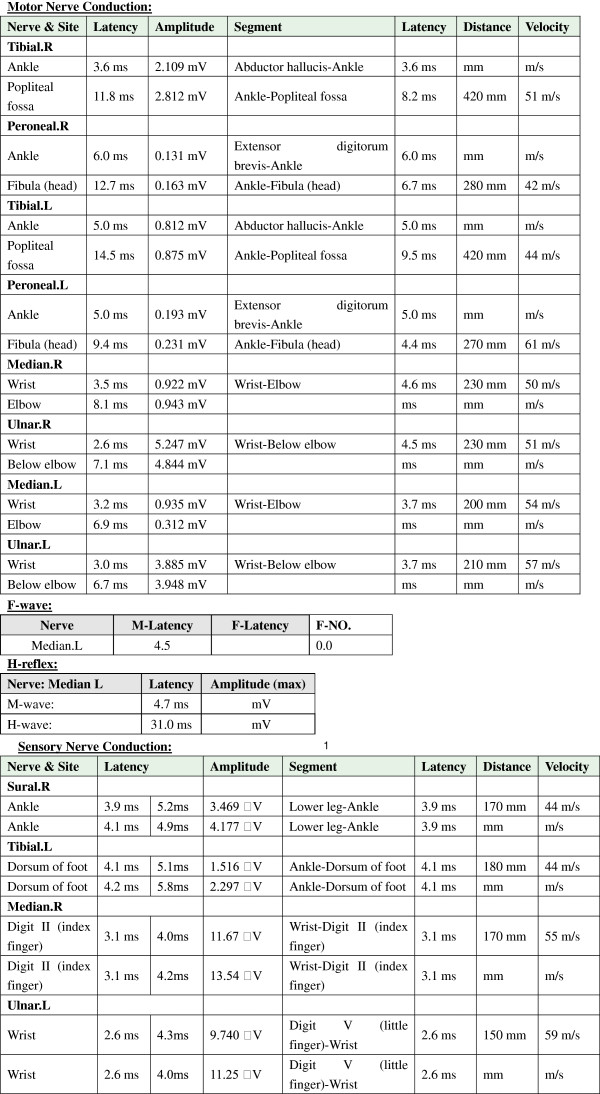
**Image of nerve conduction study data.** Motor nerve conduction study showed normal velocity in the four limbs but variable decreased amplitude at the median, ulnar, tibial, and peroneal nerves on both sides. The right peroneal nerve showed slightly prolonged latency. The sensory nerve conduction study was otherwise normal except for the decreased amplitude at the left tibial nerve. Left median F-wave could not be elicited, but H-wave was evoked normally.

A 20-year-old male, suffered from cough, rhinorrhea, wheezing and dyspnea after exposure to rainy environmental conditions. He was diagnosed with asthma in childhood. Positive family history of asthma included his mother and three elder sisters. Following treatment with inhaled corticosteroids, the symptoms abated over the next three days. Nine days after the onset of his asthma exacerbation, he developed an unsteady gait (day 1). The symptoms persisted, and on day 3 he developed blurred vision, dizziness, and nausea. On day 12, he became intermittently drowsy, however, he could be aroused by noxious stimulation. Six days later (day 18), he experienced an episode of tonic-clonic seizures. This episode brought him to the attention of the neurological team. On neurological examination he was fully conscious, had a wide-based gait, and was unable to stand on one foot. Limitations of lateral gaze in the left eye and vertical gaze in both eyes were observed. Motor and sensory functions were intact. Brain computed tomography (CT) and cerebrospinal fluid (CSF) examination showed no abnormalities. Electroencephalography (EEG) showed a 4–6 cycle per second slow wave pattern diffusely, and a 22–26 cycle per second waveform predominantly over centroparietal area, bilaterally (Figure 1). Brainstem encephalitis was tentatively diagnosed, and the patient was given intravenous dexamethasone (10 mg per day) for treatment. Despite the treatment, the patient’s symptoms deteriorated, his level of consciousness varied from occasional drowsiness to lethargy, and on day 21, he developed quadriplegia. On the Medical Research Council (MRC) scale, his muscle strength was grade one for all limbs. Triceps brachii and biceps brachii reflexes were decreased bilaterally, and brachioradialis reflexes were absent bilaterally. Patellar and achilles reflexes were also absent bilaterally. Plantar reflexes were equivocal. CSF examination showed albuminocytologic dissociation with 64 mg/mL protein and 2/μL cells. Serologic and CSF screenings for IgM antibodies against cytomegalovirus (CMV), Herpes Simplex Virus I (HSV-I), Coxsackie virus (CV), Measles virus (MV), Epstein-Barr virus (EBV), as well as EBV viral capsid antigen (VCA) IgA were all negative. Serologic analysis of antibodies against ganglioside complexes (anti-GDIa, anti-GDIb, anti-GM1, anti-GM2, anti-GM3, anti-GQIb and anti-GTIb antibodies) were negative. Serum and CSF examination, revealed significantly elevated IgE levels in both the serum (14.4 mg/L, normal range: 0.1-0.9 mg/L) and CSF (0.046 mg/L, normal range: undetectable), whereas, IgA, IgM and IgG were within the normal range. Nerve conduction study (NCS) revealed peripheral nerve abnormalities characterized by axonal damage (Figure 2). Both motor conduction velocity and sensory conduction velocity were normal in the four limbs. Motor nerve conduction study showed variable decreased amplitude at the median, ulnar, tibial, and peroneal nerves on both sides. The right peroneal nerve showed slightly prolonged latency. Sensory nerve conduction study recorded decreased amplitude at the left tibial nerve, but the right sural nerve, right median nerve and left ulnar nerve were relatively spared. Left median F-wave could not be elicited, but H-wave was evoked normally. Overall, the NCS findings were consistent with a diagnosis of GBS with predominant axonal damage.

## Results

The patient was diagnosed with BBE and concurrent MFS and GBS. The patient was in a hypersensitive state as evidenced by his increased serum and CSF IgE levels and precipitating asthma symptoms, thus intravenous dexamethasone (20 mg per day) was used. The dose of dexamethasone was reduced by half five days later, then after another five days, switched to oral prednisone and tapered. The patient improved gradually; by day 37, he could speak hoarsely and feed himself. His eye movements were sufficient, but demonstrated horizontal nystagmus. Symmetrical limb weakness registered 2–3 in the arms and 1–2 in the legs on the MRC scale. Deep tendon reflexes were present, but reduced in all 4 limbs. A follow-up visit by telephone, on day 132, found the patient still recovering. His nystagmus and ataxia had disappeared, the muscle strength of his arms had recovered, he could walk around with support, and the only problem remaining was bilateral leg weakness.

## Conclusions

BBE, MFS and GBS are considered to form a continuous clinical spectrum with variable central nervous system (CNS) and peripheral nervous system (PNS) involvement. Diagnosis of each disorder relies primarily on clinical presentation and physical examination (Table 1). According to the diagnostic criteria put forward by Odaka et al. [2], BBE constitutes a clinical entity characterized by acute ophthalmoplegia, ataxia, disturbance of consciousness or brisk reflexes. Abnormal lesions on MRI were found in about 30% of patients, and EEG presented slow-wave activity in the θ to δ range indicating involvement of CNS. Albuminocytological dissociation in the CSF and anti-GQ1b IgG antibody in serum are frequently detected. GBS is an acute demyelinating polyneuropathy manifested as progressive and symmetrical limb weakness for up to four weeks, accompanied by loss of deep tendon reflexes. Supportive evidences include relatively mild sensory loss, cranial neuropathy, and neurophysiological changes involving demyelination or axonal damage in peripheral nerves and spinal roots [6]. MFS is a rare variant of GBS, featured by a clinical triad of ophthalmoplegia, ataxia and areflexia. Albuminocytological dissociation is present in most cases of GBS and MFS [7].

**Table 1:** Diagnostic criteria for Bickerstaff’s brainstem encephalitis (BBE), Miller Fisher syndrome (MFS) and Guillain-Barré syndrome (GBS)

Disorder	Featured characteristics	Supportive evidence
BBE	Acute ophthalmoplegia, ataxia, disturbed consc-iousness or hypereflexia	Abnormal lesions on brain MRI; EEG showing abnormal slow-wave activity; anti-GQ1b IgG antibody in serum; Albuminocytological dissociation in the CSF
MFS	Acute ophthalmoplegia, ataxia and areflexia	Albuminocytological dissociation in the CSF; anti-GQ1b IgG antibody in serum
GBS	Acute symmetrical limb weakness and areflexia	Albuminocytological dissociation in the CSF; relatively mild sensory loss; cranial neuropathy; EMG showing demyelination or axonal damage in peripheral nerves and spinal roots

*CSF* cerebrospinal fluid; *MRI* magnetic resonance imaging; *EMG* electromyogram; *EEG* electroencephalogram.

Odaka and his colleagues [8] reviewed clinical profiles and laboratory findings in 62 cases of BBE, and found a high proportion (37 out of 62, 60%) of patients with concurrent GBS. These GBS syndromes were predominantly characterized by axonal damage. Some patients had additional MFS, suggesting that the three conditions are closely related. Our patient initially presented with ataxia and ophthalmoplegia, and later developed consciousness disturbances. As the disease progressed, his deep tendon reflexes reduced to areflexia, and he developed quadriplegia. At this time, the CSF began to show albuminocytologic dissociation. Based on the course of illness, the patient fits a diagnosis of a combined BBE, MFS and GBS syndrome. An axonal form of GBS revealed by electromyogram (EMG) in our patient supports the idea that a considerable number of BBE patients have a concurrent axonal Guillain-Barré syndrome [8].

Prior to the occurrence of BBE, our patient had a cough and rhinorrhea. These symptoms were quickly followed by wheezing and dyspnea, diagnosed as an episode of bronchial asthma. Further examinations of CMV, HSV-I, CV, MV and EBV in serum and CSF showed negative results, and serum antibodies against ganglioside complexes were also negative. Our findings support neither the proposal of BBE, MFS and GBS as an anti-GQ1b syndrome [2], nor the idea of an anti-GM1-mediated process for this clinical spectrum [3]. A special phenomenon associated with our patient is his past history of bronchial asthma and positive family history. Twenty-one days after the onset of BBE, his serum and CSF immunoglobulin showed normal concentrations of IgA, IgM and IgG, but significantly elevated IgE levels. Fifteen-fold increase of serum IgE level in the patient suggests that the asthma episode precipitated the combined BBE, MFS and GBS syndrome. In fact, asthma patients usually have a higher serum IgE level at the remission stage compared with healthy people [9], and IgE mediated autoallergy has been implicated in various autoimmune disorders such as rheumatoid arthritis, bullous pemphigoid and chronic spontaneous urticaria [10,11]. Most recently, Calenoff reported that IgE-mediated mast cell degranulation was possibly involved in the pathogenesis of multiple sclerosis, a well-known CNS autoimmune disease [12]. The association of asthma with BBE in our patient was indirectly supported by his response to steroid treatment.

Recently, an intriguing development in the field of asthma is its relationship with autoimmune disease. Asthma had long been considered a classic allergy disorder, thought to be mechanistically, distinctly different from autoimmune disorders. However, during the past several years, multiple studies have reported a high proportion of asthma patients with coexisting autoimmune diseases: type 1 diabetes, rheumatoid arthritis, Crohn’s disease and Addison’s disease were most frequently reported [13]. Based on these findings, a common pathogenetic effector pathway has been suggested, and mast cells, T cells, and cytokines are all potential candidates as key regulators of the immune response in both asthma and autoimmune conditions [4].

An autoimmune mechanism is most probably involved in the pathogenesis of asthma, and the present case suggests that the clinical spectrum composed of BBE, MFS and GBS is also associated with an autoimmune mechanism [2]. Recently, increased incidence of antinuclear antibodies and autoantibodies against bronchial epithelial or endothelial antigens have been found in asthma patients, such as autoantibodies against cytokeratin-18, alpha-enolase and IgE [14,15]. In addition, common loci of single nucleotide polymorphisms (SNPs) have been found to alter the risk for asthma, type 1 diabetes, primary biliary cirrhosis, and Crohn disease [5]. Therefore, although serum antibodies against ganglioside complexes were negative in our patient, the possibility exists that other antibody-mediated autoimmune responses or SNPs facilitated susceptibility and induced a continuous morbidity of BBE, MFS and GBS.

## Consent

Written informed consent was obtained from the patient for publication of this Case report and any accompanying images. A copy of written consent is available for review by the Editor-in-Chief of this journal.

## Abbreviations

BBE, Bickerstaff’s brainstem encephalitis; MFS, Miller Fisher syndrome; GBS, Guillain-Barré syndrome; CT, Computed tomography; CSF, Cerebrospinal fluid; EEG, Electroencephalography; MRC, Medical Research Council; CMV, Cytomegalovirus; HSV-I, Herpes Simplex Virus I; CV, Coxsackie virus; MV, Measles virus; EBV, Epstein-Barr virus; VCA, Viral Capsid antigen; NCS, Nerve conduction study; CNS, Central nervous system; PNS, Peripheral nervous system; EMG, Electromyography; MRI, Magnetic resonance imaging; SNPs, Single nucleotide polymorphisms.

## Competing interests

The authors declare that they have no competing interest.

## Authors’ contribution

CH made substantial contribution to case study and data collection. YW also made substantial contribution to the study by data analysing, manuscript drafting and revision. JJ and XJ gave their expertise in study designing and manuscript construction. VF and YD helped in reviewing the content critically and in language correction. WS helped to analyze and interpretate the electrophysiological data of the patient. JX helped to collect and trim the nerve conduction study data. YXS was responsible for the whole study including case analysis, study organizing and manuscript drafting. All authors have read and approved the final version of the manuscript.

## Authors’ information

Yuan Wang as co-first author.
